# Molecular characterization of thalassemia and hemoglobinopathy in Southeastern China

**DOI:** 10.1038/s41598-019-40089-5

**Published:** 2019-03-05

**Authors:** Hailong Huang, Liangpu Xu, Meihuan Chen, Na Lin, Huili Xue, Lingji Chen, Yan Wang, Deqin He, Min Zhang, Yuan Lin

**Affiliations:** Prenatal Diagnosis Center of Fujian Provincial Maternity and Children’s Hospital, Affiliated Hospital of Fujian Medical University, Fujian Provincial Key Laboratory for Prenatal Diagnosis and Birth Defect, Fuzhou, 350001 Fujian Province China

## Abstract

Thalassemia and hemoglobinopathy are two common inherited disorders, which are highly prevalent in southern China. However, there is little knowledge on the genotypes of thalassemia and hemoglobinopathy in Southeastern China. In this study, we present a large-scale genetic detection and molecular characterization of thalassemia and hemoglobinopathy in Fujian province, Southeastern China. A total of 189414 subjects screened for thalassemia were recruited, and the hemoglobin components and levels were investigated. Furthermore, suspected common thalassemia was identified, and the suspected rare forms of common thalassemias and hemoglobinopathy were detected. Among the total subjects screened, the overall prevalence of thalassemia and hemoglobinopathy was 6.8% and 0.26%, and rare α-thalassemia genotypes HKαα, –^THAI^/αα and −α^27.6^/αα, and novel β-thalassemia gene mutations CD90(G → T) and IVS-I-110(G > A) were identified. Additionally, Hb Q-Thailand hemoglobinopathy and five other types of hemoglobinopathies (Hb New York, Hb J-Bangkok, Hb G-Taipei, Hb G-Coushatta and Hb Maputo) were found. The results of this 10-year large-scale study demonstrate high prevalence of thalassemia with complicated gene mutations in Southeastern China, which provides valuable baseline data for genetic counseling and prenatal diagnosis. In addition to detection of common thalassemia genes, detection of rare thalassemia genotypes and hemoglobinopathies is recommended.

## Introduction

Thalassemia is an inherited autosomal recessive blood disorder characterized by abnormal hemoglobin production^1^. Due to genetic defects, there is reduced or absent synthesis of one or several globin peptide chains, resulting in haemolytic anemia^1^. Worldwide, thalassemias mainly occur in Mediterranean, Middle East, Indian subcontinent and Southeast Asia^2^. In China, this inherited blood disorder is highly prevalent in southern regions, and is most common in Guangdong, Guangxi, Fujian, Yunnan, Guizhou and Sichuan provinces^3–8^. Currently, detection of carriers and prenatal genetic diagnosis are the only effective interventions to prevent the birth of babies with thalassemias major and intermediate, due to lack of effective treatments for thalassemia major^9^.

Fujian province, which is located along the southeastern coastal regions of China, is highly prevalent for thalassemias^8^. To date, however, there is no large-scale analysis pertaining to the prevalence and molecular characterization of thalassemia and hemoglobinopathy in this region, which greatly influences the genetic counseling of thalassemia and hemoglobinopathy and prenatal diagnosis. The present large-scale study was therefore designed with aims to detect the thalassemia gene mutations and characterize the genotypes of rare forms of thalassemias and hemoglobinopathy in Fujian province, Southeastern China during the 10-year period from 2008 through 2017. Such a study may provide baseline data for genetic counseling and prenatal diagnosis of thalassemia and hemoglobinopathy and prenatal diagnosis in this region, so as to prevent the birth of babies with thalassemias major.

## Methods

### Ethical statement

This study was reviewed and approved by the Ethics Review Committee of Fujian Provincial Maternity and Children Hospital. Signed informed consent was obtained from all participants following a detailed description of the purpose of the study. All experiments were performed in accordance with relevant guidelines and regulations.

### Study subjects

A total of 189414 subjects that were screened for thalassemia at the Outpatient Department of Fujian Provincial Maternity and Children’s Hospital during the period from January 2008 through July 2017 were recruited. The subjects had a mean age of 27 years (range, 1 to 69 years), and came from 9 cities across the province. All subjects had no genetic relationships.

### Screening of thalassemia

Peripheral blood samples were collected from each subject and anticoagulated with EDTA-K2. Approximately 2 mL of the anticoagulated blood samples were used for analysis of blood cell parameters on a Sysmex XN-2000 automatic hematology analyzer (Sysmex; Shanghai, China), and the hemoglobin components and levels were analyzed using high-performance liquid chromatography (HPLC) with a VARIANT II TURBO Hemoglobin Testing System (Bio-Rad Laboratories, Inc.; Hercules, CA, USA). Positive thalassemia screening was defined as a mean corpuscular volume (MCV) of <80 fL, a mean corpuscular hemoglobin (MCH) concentration of <27 pg, and/or hemoglobin A2 (HbA2) of >4.0% and/or fetal hemoglobin (HbF) of >2.0% and HbA2 of <2.5%^1^. All patients positive for thalassemia screening were subjected to genetic testing of thalassemias.

### Genotyping

Genomic DNA was extracted from the peripheral blood samples using a genomic DNA isolation kit (Qiagen; Hilden, Germany) following the manufacturer’s instructions. The three common deletional α-thalassemias were detected using Gap-PCR with the thalassemia gene detection kit (Shenzhen Yishengtang Biological Products Co., Ltd.; Shenzhen, China)^10^, and detection of the point mutations in the three non-deletional α-thalassemias and genetic diagnosis of β-thalassemia were done using reverse dot-blot hybridization (RDB) with the thalassemia gene detection kit (Shenzhen Yishengtang Biological Products Co., Ltd.; Shenzhen, China) following the manufacturers’ instructions^11^.

The deletional fragments of the α-thalassemia genes were detected using the SALSA multiplex ligation-dependent probe amplification (MLPA) P027.B1 assay (MRC Holland; Amsterdam, The Netherlands), and verified by means of Gap-PCR and breakpoint sequencing^12^. Suspected deletional β-thalassemias were detected with the SALSA MLPA P102-B1 HBB assay (MCR-Holland; Amsterdam, The Netherlands), and definitively diagnosed using Gap-PCR with the deletional β-thalassemia gene detection kit (Shenzhen Yaneng Biological Enterprise Co., Ltd.; Shenzhen, China). The detection results were processed using the software MRC-Coffalyser version 9.4 (MRC Holland; Amsterdam, The Netherlands).

For suspected rare types of α- and β-thalassemias, the full-length α- and β-globin genes were amplified using PCR assay and checked. The purified PCR products were subjected to direct sequencing with an ABI 3100 DNA Sequencer (Applied Biosystems; Foster City, CA, USA).

### Data analysis

All data were entered into and managed using Microsoft Excel 2007 (Microsoft; Redmond, WA, USA). The gene mutations, frequency and spectrum of thalassemia and hemoglobinopathy were analyzed with a descriptive method.

## Results

### Prevalence of thalassemia and hemoglobinopathy

Among the 189414 subjects screened for thalassemias, there were 31118 cases positive for screening, including 13400 cases definitively diagnosed with thalassemias, 7966 cases with iron deficiency and 9752 cases with unknown causes. The overall prevalence of thalassemia (12883 cases) was 6.8%, and the prevalence of α-thalassemia (9173 cases), β-thalassemia (3542 cases) and concurrent α- and β-thalassemias (186 cases) was 4.84%, 1.87% and 0.1%, respectively. In addition, the prevalence of hemoglobinopathy (499 cases) was 0.26% (Table 1).

**Table 1:** Prevalence of thalassemia and hemoglobinopathy in Fujian province, Southeastern China.

Disorder	No. patients detected	Constituent ratio (%)	Prevalence (%)
α-thalassemia	9173	68.46	4.84
β-thalassemia	3542	26.43	1.87
Concurrent α- and β-thalassemias	186	1.39	0.1
Hemoglobinopathy	499	3.72	0.26
Total	13400	100	7.07

### Genotypes of common and rare types of α-thalassemias

Among the 9173 cases detected with α-thalassemias, there were 8949 cases with common α-thalassemias (97.56%) and 224 cases with rare forms of α-thalassemias (2.44%). In common α-thalassemias, a total of 6 mutated genes were detected, including three deletional and three non-deletional mutations. The most frequent deletional mutation was seen in the genotype–^SEA^/αα (66.34%), followed by in genotypes −α^3.7^/αα (18.39%) and −α^4.2^/αα (4.45%), and the three most frequent non-deletional mutation were detected in the genotypes α^QS^α/αα (2.67%), α^CS^α/αα and α^WS^α/αα (both 1.16%). In rare forms of α-thalassemias, the two most common genotypes were–^THAI^/αα (1.06%) and HKαα/αα (0.97%) (Table 2).

**Table 2:** Genotyping of α-thalassemia in Fujian province, Southeastern China.

	Genotype	Phenotype	No. patients detected	Constituent ratio (%)
Common α-thalassemia	−α^3.7^/αα	α^+^/α	1687	18.39
	−α^4.2^/αα	α^+^/α	408	4.45
	–^SEA^/αα	α^0^/α	6085	66.34
	α^CS^α/αα	α^+^/α	106	1.16
	α^QS^α/αα	α^+^/α	245	2.67
	α^WS^α/αα	α^+^/α	106	1.16
	–^SEA^/-α^3.7^	α^0^/α^+^	139	1.52
	–^SEA^/-α^4.2^	α^0^/α^+^	56	0.61
	α^CS^α/–^SEA^	α^+^/α^0^	22	0.24
	α^WS^α/–^SEA^	α^+^/α^0^	7	0.08
	α^QS^α/–^SEA^	α^+^/α^0^	13	0.14
	−α^3.7^/−α^4.2^	α^+^/α^+^	10	0.11
	−α^4.2^/−α^4.2^	α^+^/α^+^	10	0.11
	−α^3.7^/−α^3.7^	α^+^/α^+^	30	0.33
	α^CS^α/−α^4.2^	α^+^/α^+^	2	0.02
	α^CS^α/−α^3.7^	α^+^/α^+^	5	0.05
	α^QS^α/α^QS^α	α^+^/α^+^	2	0.02
	α^QS^α/−α^3.7^	α^+^/α^+^	6	0.06
	α^QS^α/−α^4.2^	α^+^/α^+^	3	0.03
	α^WS^α/−α^4.2^	α^+^/α^+^	2	0.02
	α^WS^α/−α^3.7^	α^+^/α^+^	5	0.05
Subtotal		—	8949	97.56
Rare α-thalassemia	HKαα/αα	α^+^/α	89	0.97
	HKαα/–^SEA^	α^+^/α^0^	7	0.08
	HKαα/−α^3.7^	α^+^/α^+^	18	0.20
	–^SEA^/−α^27.6^	α^0^/α^+^	3	0.03
	−α^27.6^/αα	α^+^/α	5	0.05
	–^THAI^/αα	α^0^/α	97	1.06
	−α^3.7^/–^THAI^	α^+^/α^0^	2	0.02
	−α^3.7^/anti^4.2^	α^+^/α^+^	3	0.03
Subtotal		—	224	2.44
Total		—	9173	100

α^0^ indicates absent synthesis of α-globin peptide chain; α^+^ indicates reduced synthesis of α-globin peptide chain; α indicates no mutation.

### Genotypes of common and rare types of β-thalassemias

**Figure 1:**
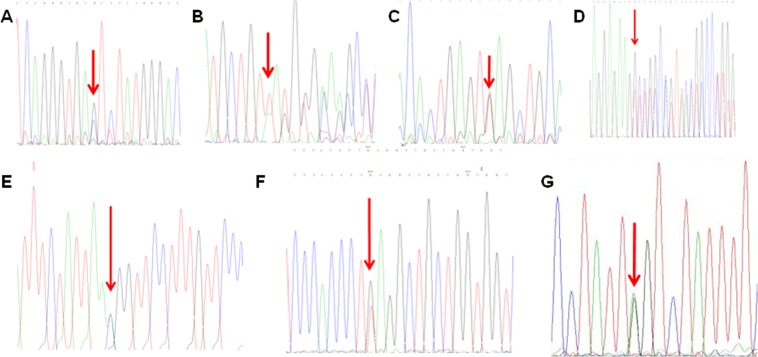
DNA sequencing reveals hemoglobin variants in 7 rare forms of β-thalassemia. (**A**) CD90(G > T); (**B**) CD54-58(−TATGGGCAACCCT); (**C**) IVS-II-5(G > C); (**D**) CD8-9(+G); (**E**), TermCD + 32(A > C); (**F**) IVS-I-128 (T > G); (**G**) IVS-I-110(G > A).

Among the 3542 cases detected with β-thalassemias, there were 3514 cases with common β-thalassemias (99.21%) and 28 cases with rare forms of β-thalassemias (0.79%). In common β-thalassemias, the three most frequent mutations were seen in genotypes β^IVS-2-654(C→T)^/β^N^ (41.95%), β^CD41-42(-TCTT)^/β^N^ (30.26%) and β^CD17(A→T)^/β^N^ (12.25%). In rare forms of β-thalassemias, three deletional mutations (Southeast Asian hereditary persistence of fetal hemoglobin (SEA-HPFH), Chinese G_γ_^+^(A_γ_δβ^0^) and Taiwan deletion) and 10 rare β-thalassemia mutations were detected (Table 3, Fig. 1).

**Table 3:** Genotyping of β-thalasse vcmia in Fujian province, Southeastern China.

	Genotype	Phenotype	No. patients detected	Constituent ratio (%)
Common β-thalassemia	β^IVS-2-654(C→T)^/β^N^	β^+^/β^N^	1486	41.95
	β^CD41-42(-TCTT)^/β^N^	β^0^/β^N^	1072	30.26
	β^CD17(A→T)^/β^N^	β^0^/β^N^	434	12.25
	β^−28(A→G)^/β^N^	β^+^/β^N^	194	5.48
	βCD27-28(+C)/β^N^	β^0^/β^N^	106	2.99
	β^CD43(G→T)^/β^N^	β^0^/β^N^	21	0.59
	β^Int(ATG→AGG)^/β^N^	β^0^/β^N^	11	0.31
	β^CD26(G→A)^/β^N^	βE	75	2.11
	β^−29(A→G)^/β^N^	β^+^/β^N^	9	0.25
	β^IVS-1-1(G→T)^/β^N^	β^0^/β^N^	3	0.08
	β^IVS-1-5(G→T)^/β^N^	β^+^/β^N^	4	0.11
	β^CD71-72(+A)^/β^N^	β^0^/β^N^	26	0.73
	β^CD14-15(+G)^/β^N^	β^0^/β^N^	3	0.08
	β^CAP+40-+43(-AAAC)^/β^N^	β^+^/β^N^	7	0.2
	β^−30(T→C)^/β^N^	β^+^/β^N^	2	0.06
	β^IVS-2-654(C→T)^**/β**^IVS-2-654(C→T)^	β^+^/β^+^	12	0.34
	β^IVS-2-654(C→T)^/**β**^CD41-42(-TCTT)^	β^+^/β^0^	13	0.37
	β^IVS-2-654(C→T)^/**β**^CD17(A→T))^	β^+^/β^0^	6	0.17
	β^IVS-2-654(C→T)^/**β**^−28(A→G)^	β^+^/β^+^	1	0.03
	β^IVS-2-654(C→T)^/**β**^CD27-28(+C)^	β^+^/β^0^	2	0.06
	β^IVS-2-654(C→T)^/**β**^CD26(G→A)^	β^+^/βE	1	0.03
	β^IVS-2-654(C→T)^/**β**^−29(A→G)^	β^+^/β^+^	1	0.03
	β^IVS-2-654(C→T)^/**β**^CD43(G→T)^	β^+^/β^0^	1	0.03
	β^CD41-42(-TCTT)^/**β**^CD41-42(-TCTT)^	β^0^/β^0^	5	0.14
	β^CD41-42(-TCTT)^/**β**^CD17(A→T)^	β^0^/β^0^	2	0.06
	β^CD41-42(-TCTT)^/**β**^−28(A→G)^	β^0^/β^+^	3	0.08
	β^CD41-42(-TCTT)^/**β**^CD71-72(+A)^	β^0^/β^0^	1	0.03
	β^CD17(A→T)^/**β**^CD17(A→T)^	β^0^/β^0^	1	0.03
	β^CD17(A→T)^/**β**^−28(A→G)^	β^0^/β^+^	2	0.06
	β^CD17(A→T)^/**β**^CD26(G→A)^	β^0^/βE	1	0.03
	β^−28(A→G)^/**β**^−28(A→G)^	β^+^/β^+^	4	0.11
	β^CD26(G→A)^/**β**^CD26(G→A)^	βE/βE	4	0.11
	β^−29(A→G)^/**β**^CD71-72(+A)^	β^+^/β^0^	1	0.03
Subtotal		—	3514	99.21
Rare β-thalassemia	Deletional SEA-HPFH	β^0^/β^N^	6	0.17
	Chinese G_γ_^+^(A_γ_δβ^0^)	β^0^/β^N^	6	0.17
	Taiwanese deletion	β^0^/β^N^	1	0.03
	β^+22(G→A)^/β^N^	β^+^/β^N^	2	0.06
	β^CD30(G→A)^/β^N^	β^0^/β^N^	2	0.06
	β^CD90(G→T)^/β^N^	β^0^/β^N^	2	0.06
	β^CD54-58(−TTATGGGCAACCC)^/β^N^	β^0^/β^N^	2	0.06
	β^CD36(-C)^/β^N^	β^0^/β^N^	2	0.06
	β^IVS-2-5((G>C)^/β^N^	β^+^/β^N^	1	0.03
	β^CD8-9(+G)^/β^N^	β^0^/β^N^	1	0.03
	β^TermCD+32(Aå C)^/β^N^	β^+^/β^N^	1	0.03
	β^IVS-I-128(Tå G)^/β^N^	β^+^/β^N^	1	0.03
	β^IVS-I-110(Gå A)^/β^N^	β^+^/β^N^	1	0.03
Subtotal		—	28	0.79
Total		—	3542	100

β^0^ indicates absent synthesis of β-globin peptide chain; β^+^ indicates reduced synthesis of β-globin peptide chain; N indicates no mutation; SEA-HPFH, Southeast Asian hereditary persistence of fetal hemoglobin.

### Genotypes of concurrent α- and β-thalassemias

Among the 186 cases with concurrent α- and β-thalassemias, there were 28 genotypes detected in the 173 cases with common forms of concurrent α- and β-thalassemias (93.01%) and 2 genotypes detected in the 13 cases with rare forms (6.99%) (Table 4).

**Table 4:** Genotyping of concurrent α- and β-thalassemias in Fujian province, Southeastern China

	Genotype	Phenotype	No. patients detected	Constituent ratio (%)
Commonconcurrent α- and β-thalassemias	β^IVS-2-654(C→T)^/β^N^/–^SEA^/αα	β^+^/β^N^α^0^/α	23	12.37
	β^IVS-2-654(C→T)^/β^N^/-α^3.7^/αα	β^+^/β^N^α^+^/α	24	12.9
	β^IVS-2-654(C→T)^/β^N^/-α-α^4.2^/αα	β^+^/β^N^α^+^/α	7	3.76
	β^IVS-2-654(C→T)^/β^N^/α^QS^α/αα	β^+^/β^N^α^+^/α	6	3.23
	β^IVS-2-654(C→T)^/β^N^/α^WS^α/αα	β^+^/β^N^α^+^/α	1	0.54
	β^IVS-2-654(C→T)^/β^N^/α^CS^α/αα	β^+^/β^N^α^+^/α	1	0.54
	β^IVS-2-654(C→T)^/β^N^/–^SEA^/-α^3.7^	β^+^/β^N^α^0^/α^+^	1	0.54
	β^CD41-42(-TCTT)^/β^N^/–^SEA^/αα	β^0^/β^N^α^0^/α	24	12.9
	β^CD41-42(-TCTT)^/β^N^/-α^3.7^/αα	β^0^/β^N^α^+^/α	26	13.98
	β^CD41-42(-TCTT)^/β^N^/-α^4.2^/αα	β^0^/β^N^α^+^/α	6	3.23
	β^CD41-42(-TCTT)^/β^N^/–^THAI^/αα	β^0^/β^N^α^0^/α	1	0.54
	β^CD41-42(-TCTT)^/β^N^/α^WS^α/αα	β^0^/β^N^α^+^/α	5	2.69
	β^CD41-42(-TCTT)^/β^N^/α^CS^α/αα	β^0^/β^N^α^+^/α	2	1.08
	β^CD41-42(-TCTT)^/β^N^/-α^4.2^/α^WS^α	β^0^/β^N^α^+^/α^+^	1	0.54
	β^CD27-28(+C)^/β^N^/–^SEA^/αα	β^0^/β^N^α^0^/α	1	0.54
	β^CD27-28(+C)^/β^N^/-α^3.7^/αα	β^0^/β^N^α^+^/α	1	0.54
	β^−28(A→G)^/β^N^/–^SEA^/αα	β^+^/β^N^α^0^/α	4	2.15
	β^CD17(A→T)^/β^N^/–^SEA^/αα	β^0^/β^N^α^0^/α	16	8.6
	β^CD17(A→T)^/β^N^/-α^3.7^/αα	β^0^/β^N^α^+^/α	8	4.30
	β^CD17(A→T)^/β^N^/-α^4.2^/αα	β^0^/β^N^α^+^/α	3	1.61
	β^CD17(A→T)^/β^N^/–^THAI^/αα	β^0^/β^N^α^0^/α	1	0.54
	β^CD17(A→T)^/β^N^/α^QS^α/αα	β^0^/β^N^α^+^/α	2	1.08
	β^CD17(A→T)^/β^N^/α^CS^α/αα	β^0^/β^N^α^+^/α	1	0.54
	β^CD43(G→T)^/β^N^/–^SEA^/αα	β^0^/β^N^α^0^/α	1	0.54
	β^IVS-1-1(G→T)^/β^N^/-α^4.2^/αα	β^0^/β^N^α^+^/α	1	0.54
	β^CAP+40-+43(-AAAC)^/β^N^/–^SEA^/αα	β^+^/β^N^α^0^/α	2	1.08
	β^CAP+40-+43(-AAAC)^/β^N^/-α^3.7^/αα	β^+^/β^N^α^+^/α	1	0.54
	β^−28(A→G)^/β^N^/-α^3.7^/αα	β^+^/β^N^α^+^/α	3	1.61
	Subtotal	—	173	93.01
Rare concurrent α- and β-thalassemias	β^CD41-42(-TCTT)^/β^N^/HKαα/αα	β^0^/β^N^α^+^/α	10	5.38
	β^IVS-2-654(C→T)^/β^N^/HKα/αα	β^+^/β^N^α^+^/α	3	1.61
	Subtotal	—	13	6.99
Total		—	186	100.00

α^0^ indicates absent synthesis of α-globin peptide chain; α^+^ indicates reduced synthesis of α-globin peptide chain; β^0^ indicates absent synthesis of β-globin peptide chain; β^+^ indicates reduced synthesis of β-globin peptide chain; α and N indicate no mutation.

### Genotypes of hemoglobinopathy

**Figure 2:**
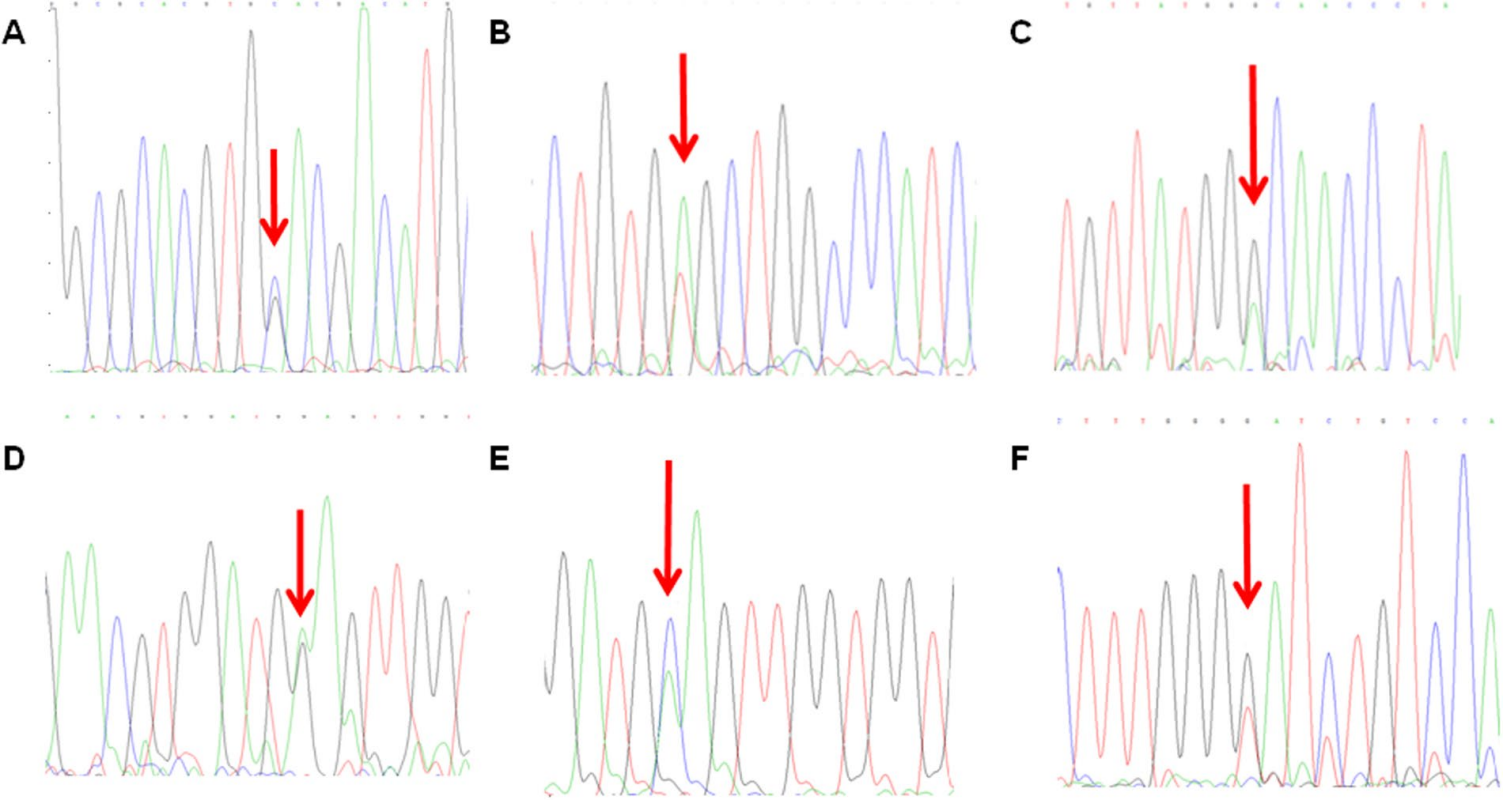
DNA sequencing reveals hemoglobin variants in 6 rare forms of hemoglobinopathy. (**A**) CD74(GAC > CAC),Hb Q-Thailand; (**B**) CD113(GTG > GAG), Hb New York; (**C**) CD56(GGC > GAC), Hb J-Bangkok; (**D**) CD22(GAA > GGA), Hb G-Taipei; (**E**) CD22(GAA > GCA), Hb G-Coushatta; (**F**) CD47(GAT > TAT), Hb Maputo.

We detected an Hb Q-Thailand/−α^4.2^ hemoglobinopathy induced by α-globin gene mutation in 141 cases with, and five other types of hemoglobinopathies induced by β-globin gene mutation, including Hb New York (CD113(GTG > GAG)) in 317 cases, Hb J-Bangkok (CD56(GGC > GAC)) in 35 cases, Hb G-Taipei (CD22(GAA > GGA)) in one case, Hb G-Coushatta (CD22(GAA > GCA)) in 4 cases and Hb Maputo (CD47(GAT > TAT)) in one case (Table 5, Fig. 2).

**Table 5:** Detection and phenotypes of hemoglobinopathy in Fujian province, Southeastern China.

Hemoglobin variant	Phenotype	No. patients detected	Constituent ratio (%)
Hb Q-Thailand/-α^4.2^	HbVar/α^+^	141	28.26
Hb New York (CD113(GTG > GAG))	HbVar	317	63.53
Hb J-Bangkok (CD56(GGC > GAC))	HbVar	35	7.01
Hb G -Taipei (CD22(GAA > GGA))	HbVar	1	0.2
Hb G-Coushatta(CD22(GAA > GCA))	HbVar	4	0.8
Hb Maputo(CD47(GAT > TAT))	HbVar	1	0.2
Total	—	499	100

## Discussion

Epidemiological data have shown that thalassemia is highly prevalent in Fujian province, southeastern China^7,8^; however, there are few studies reporting the thalassemia genotypes, and there is no knowledge on hemoglobinopathy in this region until now. In this 10-year large-scale study recruiting 189414 study populations, we detected 0.26% prevalence of hemoglobinopathy and 6.8% prevalence of thalassemia in Fujian province, southeastern China, which is higher than the 2013 sampling survey (4.41% prevalence of thalassemia)^8^.

In the present study, we detected 6.8% overall prevalence of thalassemia carriers, and 4.84%, 1.87%, 0.1% and 0.26% prevalence of α-thalassemia, β-thalassemia, concurrent α- and β-thalassemias, and hemoglobinopathy in the study subjects, respectively. Our findings confirm that thalassemia is highly prevalent in Fujian province, and suggest that screening of thalassemias should be performed to avoid the birth of babies with thalassemia major.

In this study, we detected with 15 α-thalassemia genotypes, and 97.56% were identified as common types, including 89.18% deletional mutations and 4.99% non-deletional mutations. The two most frequent deletional mutations were seen in the genotypes–^SEA^/αα (66.34%) and −α^3.7^/αα (18.39%). The spectrum of mutations detected in this study was similar to the findings observed in the neighboring provinces; however, the prevalence of genotypes differed in regions (−α^3.7^/αα was the most frequent genotype in Chongqing)^13–15^. The prevalence of rare forms of α-thalassemias was 2.44%, and–^THAI^/αα was the most common genotype (1.06%). To date, there are few studies reporting the detection of Thailand type gene mutations in thalassemias in China, and this type of mutation is only detected in Guangdong, Guangxi and Taiwan^4,5,16,17^. Like the ^–SEA^genotype, the α-thalassemia with the ^–THAI^genotype is manifested as microcytic hypochromic anemia^17^, and the Thailand-type homozygote or Thailand-type heterozygotes with SEA-type α-thalassemia are manifested as Bart’s hydrops fetalis^18^. Since Thailand-type α-thalassemia is rarely detected in southern China^16^, detection of Thailand-type gene mutation is not recommended as a routine examination, which usually results in missing diagnosis. If a spouse carries α-thalassemia genes, and another is positive for hematological phenotypes, or has had babies with thalassemia major or intermediate, but common α-thalassemia genotypes are not detected, Thailand type gene mutations are recommended for detection to avoid missing diagnosis. The prevalence of HKαα/αα allele was 0.97%. To date, there are few reports pertaining to HKαα/αα type thalassemias, which are mainly detected in Guangdong and Guangxi, China^4,5^. It has been found that the hematological manifestations is better in the HKαα/αα allele carriers than in the –α^3.7^ carriers^19^. Previous studies have demonstrated that α-globin triplication is not rare in humans^20–22^; however, the anti4.2 fragment cannot be directly detected by the currently available commercial kits^23^. The genotype −α^27.6^/αα is also firstly detected in Fujian province. If conventional commercial kits are used to screen thalassemia genotypes, some rare types of delectional mutations fail to be detected, thereby resulting in missing diagnosis. The detection and prevention of rare types of thalassemias should be therefore emphasized.

In the present study, the prevalence of common β-thalassemias was 99.21%, and β^IVS-2-654(C→T)^/β^N^ (41.95%), β^CD41-42(-TCTT)^/β^N^ (30.26%) and β^CD17(A→T)^/β^N^(12.25%) were the three most frequent genotypes. β^CD41-42(-TCTT)^/β^N^ and β^CD17(A→T)^/β^N^ were the two most frequent mutations (72.21% totally), followed by β^CD17(A→T)^/β^N^, β^−28(A→G)^/β^N^ and β^CD27-28(+C)^/β^N^, and these five genotypes consisted of 92.93% of total variants of β-thalassemias, suggesting a high genetic heterogeneity for β-thalassemia in Fujian province. Our data were different from the types of β-thalassemia gene mutations in other regions where β-thalassemia is highly prevalent. It was reported that β^CD41-42(-TCTT)^/β^N^ was the most common genotype in Guangdong, Hainan, Hunan and Jiangxi^24–27^, while β^CD17(A→T)^/β^N^ was the most common genotype in Guangxi and Chongqing, indicating region-specific prevalence of β-thalassemia genotypes^5,28^. We detected 0.79% prevalence of rare forms of β-thalassemias, in which three deletional mutations (SEA-HPFH, Chinese Chinese G_γ_^+^(A_γ_δβ^0^) and Taiwan deletion) and other 10 rare types of mutations were detected. The results demonstrate the diversity and significant genetic heterogenicities of β-thalassemias in Fujian province, and indicate that screening and detection of rare and unknown gene mutations should be emphasized. Deletional β-thalassemia is clinically characterized by elevated HbF with or without abnormal blood testing^29^. In China, there have been Southeastern Asian, Chinese and Taiwan types detected in β-thalassemias^30^. The present study, for the first time, identified 13 cases with deletional β-thalassemias in Fujian province. If the subjects carrying deletional β-thalassemias marry common β-thalassemia carriers, there is a risk of having babies with β-thalassemia intermediate or major. Rare types of deletional β-thalassemias are not detected, which may result in missing diagnosis. Our previous studies identified several novel β-thalassemia gene mutations, including codon 36 (−C) mutation that was firstly detected worldwide, and codon30 (A → G) and +22(G → A) that were firstly identified in China^31^. Then, we detected novel β-thalassemia gene mutations CD90 (G → T) and IVS-I-110 (G > A) that were firstly described in China, and CD54-58(-TTATGGGCAACCC), CD8-9(+G), IVS-2-5 (G > C) and TermCD + 32(A > C) that were firstly identified in Fujian province. The results of the present study indicate the highly prevalence of thalassemias and complicated thalassemia genotypes in Fujian province, and the identification of these rare genotypes adds valuable data into the spectrum of thalassemia gene mutations across the world.

The patients with concurrent α- and β-thalassemias are reported to have mild anemia, and this is because of reduced synthesis of α- and β-globin chains, which alleviates the imbalance induced by reduced synthesis of globin chains, leading to the alleviation of anemia^32^. In this study, we detected 0.1% prevalence of concurrent α- and β-thalassemias, and the 4 most common genotypes included β^CD41-42(-TCTT)^/β^N^/-α^3.7^/αα (26 cases), β^IVS-2-654(C→T)^/β^N^/-α^3.7^/αα (24 cases), β^CD41-42(-TCTT)^/β^N^/–^SEA^/αα (24 cases) and β^IVS-2-654(C→T)^/β^N^/–^SEA^/αα (23 cases). Although the patients with concurrent α- and β-thalassemias have mild symptoms, their offspring have a higher likelihood of developing thalassemia major than general populations, and the long-term damage is much higher. Therefore, definitive diagnosis of concurrent α- and β-thalassemias cannot be neglected.

Hemoglobinopathy and thalassemia are genetic disorders caused by aberrant hemoglobin; however, thalassemia is caused by reduced or absent synthesis of globin peptide chains^1^, while hemoglobinopathy is caused by alteration of the globin peptide chain conformation, which usually does not develop anemia^33^. In this study, we detected, for the first time, 0.26% prevalence of hemoglobinopathy carriers in Fujian province. An Hb Q-Thailand/-α^4.2^ hemoglobinopathy induced by α-globin gene mutation was identified in 141 cases, and five other types of hemoglobinopathies induced by β-globin gene mutation were detected, including Hb New York (CD113(GTG > GAG)), Hb J-Bangkok (CD56(GGC > GAC)), Hb G-Taipei (CD22(GAA > GGA)), Hb G-Coushatta (CD22(GAA > GCA)) and Hb Maputo (CD47(GAT > TAT)). Hb New York is the most common type of hemoglobinopathy, which is mutated from valine to glutamic acid in the 113th position of the β-globin peptide chain^34^. This mutation is derived from Hakka, and transmits through Hakkasan migration^35^. In addition, Hb J-Bangkok is caused by the mutation from glycine to aspartic acid in the 56th position of the β-globin peptide chain^36^. The stability of these two hemoglobinopathies is lower than HbA; however, the carriers with these two mutations don’t present anemia. Nevertheless, these two hemoglobinopathies complicated with other types of thalassemias may present clinical manifestations of thalassemias at various degrees. Therefore, definitive diagnosis of hemoglobinopathy is of great necessity.

Based on the results from this large-scale study and approximately 600000 newborns annually, it is estimated that there are 159 newborns with α-thalassemia, 70 newborns with hemoglobinopathy, and 53 cases with β-thalassemia major in Fujian province annually if no preventive or control interventions are implemented, which will cause huge social burdens. Notably, β-thalassemia major is usually complicated with severe anemia symptoms, and is still a lack of effective treatments^37^. Irregular blood transfusion is required to maintain survival throughout the life, which causes great impacts on patients’ quality of life^38^. According to the estimates of 100000 RMB annual medical costs, this will cause huge mental pain and economic burdens. Therefore, the health education and screening of thalassemias should be strengthened in regions where thalassemia is highly prevalent, and the prevention based on public education, human screening and prenatal diagnosis is critical to the prevention and control of thalassemia in Fujian province.

In summary, this long-term large-scale analysis of the common and rare thalassemia genotypes and hemoglobinopathy prevalence and genotypes add valuable data into the knowledge of thalassemias in China. The results of the present study demonstrate that thalassemias are highly prevalent in Fujian province, Southeastern China, and the thalassemia genotypes are characterized by diversity and significant genetic heterogenicities. Our findings suggest that screening and detection of rare thalassemia gene mutations should be strengthened. Our findings provide valuable baseline data for genetic counseling and prenatal diagnosis of thalassemias.

## Data Availability

All data generated during and/or analyzed during the current study are available upon request by contact the corresponding author.
